# An Embedded Laser Marking Controller Based on ARM and FPGA Processors

**DOI:** 10.1155/2014/716046

**Published:** 2014-03-18

**Authors:** Wang Dongyun, Ye Xinpiao

**Affiliations:** The Engineering School, Zhejiang Normal University, Jin Hua, Zhejiang 321005, China

## Abstract

Laser marking is an important branch of the laser information processing technology. The existing laser marking machine based on PC and WINDOWS operating system, are large and inconvenient to move. Still, it cannot work outdoors or in other harsh environments. In order to compensate for the above mentioned disadvantages, this paper proposed an embedded laser marking controller based on ARM and FPGA processors. Based on the principle of laser galvanometer scanning marking, the hardware and software were designed for the application. Experiments showed that this new embedded laser marking controller controls the galvanometers synchronously and could achieve precise marking.

## 1. Introduction

Compared with inkjet coding, laser marking has many advantages. Firstly, it is a permanent identification method, which cannot be modified. Secondly, it does not require consumables which reduce the use of cost. Thirdly, it is environment friendly with fewer emissions. With the increasingly strict requirements of product quality tracking, laser marking obtains a wide range of applications. Accordingly, more and more researchers began to study laser marking control system and its marking quality [1–5]. In 2003, Chen [6] developed a beam-steering marking system based on PC control. The utilization of 16-bit D/A card provides good positioning reliability. In 2005, Liu [7] proposed a control system design for arrayed online CO_2_ laser marker. The design and realization for a control system based on Visual C++ are discussed in this paper; the key technologies such as high-precision timing by Win32API functions, pulses output control, data accessing by register operating, and emulating design are also described. In 2005, Sabo [8] reported the advantages of digital servo amplifiers for controlling of a galvanometer based on optical scanning system. The advanced control algorithms of a digitally controlled scanning system lead to highly improved dynamics compared with an analog type where only a very limited number of parameters are used to tune the system to a certain dynamical requirement.

The existing laser marking controllers are mainly based on PC and WINDOWS operating system. It is very bulky and is inconvenient to move. Still, it cannot work outdoors or in other harsh environments. In order to solve this deficiency, this paper proposed an embedded laser marking controller based on ARM and FPGA processor.

## 2. Principles of Vectors Scanning Marking

**Figure 1:**
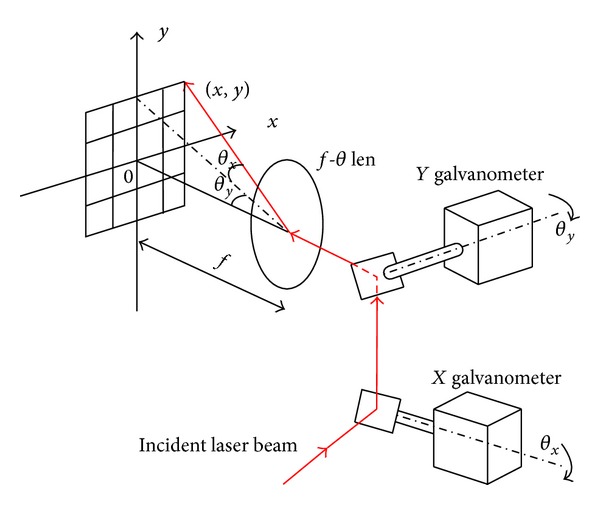
Schematic diagram of the two-dimensional laser marking system.

In the galvanometric scanning marking system, the laser generator send out a laser beam. It will be deflected by two scanning mirrors and then it will be focused on the marking plane by a f-theta lens. [1].  Figure 1 shows the principle of operation: the incident laser beam enters the system through an entrance aperture. Once inside, the laser beam irradiates onto the first reflecting mirror on the *X* scanner, and then the second mirror on the *Y* scanner deflects it. Accordingly, the laser beam would be projected on the defined image field. The galvanometric scanners combine a mirror with a servo-actuator-limited-rotation motor, which is the main component in the galvanometric scanning system. They control the laser beam onto the defined locations via turning scanning mirrors. Therefore, the mirrors can perform high-speed deflection around an axis of rotation of the galvanometric scanning motor. The single galvanometer can only deflect a laser beam along a line in an image plane. The galvanometric scanning system and the position of the laser beam must be controlled, both horizontally (*x*-axis) and vertically (*y*-axis), to create an image with two axes. *XY* scanners deflect a parallel beam in the *X*–*Y* direction.

Assuming that the length between the object to be marked and the lens is *f*, and the coordinates of the spots laser focus would be (*X*, *Y*); so, if the deflection angles of *X*∖*Y* mirror are *θ*_*x*_ and *θ*_*y*_, and if *x* = *y* = 0, then *θ*_*x*_ = *θ*_*y*_ = 0 [1]. The deflection range is generally small in the galvanometer scanning system, usually from ±5° to ±10°. In the case of small angle, the following equation can be established:

(1) $$\begin{array}{c} x=f\times tg\theta_{x}\approx f\times \theta_{x}\\ y=f\times tg\theta_{y}\approx f\times \theta_{y}. \end{array}$$

Equation (1) indicated that the coordinates of the marking point and the mirror deflection angel are linear. The maximum deflection angle corresponds with the maximum marking range.

The galvanometer is driven by a servomotor and rotates to a corresponding angle. The servomotor is controlled by voltage signal. The rotation angle is linear to the voltage level which is output by a Digital to Analog converter. As DA converter converts digital signal into a voltage signal; then laser beam can be driven to the coordinates of (*X*, *Y*) marking point by converting *X*, *Y* value to corresponding voltage signal. Consider the following:

(2) $$\begin{array}{c} \phi_{x}=k_{x}\times V_{x}\\ \phi_{y}=k_{y}\times V_{y}, \end{array}$$

where *ϕ* and *V* are the rotation angle and the control voltage signal for the *x*-axis and *y*-axis servomotors.

**Figure 2:**
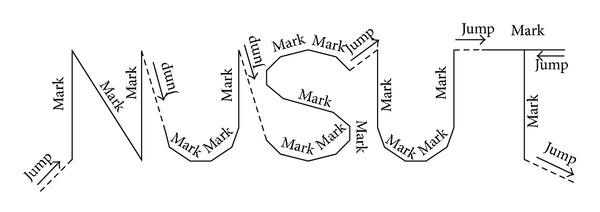
The principle of vector laser marking.

In a galvanometer scanning marking system, the marking contents should be digitized and be described with vector coordinates. Then the coordinates would be converted into voltage signal which drives the servomotor to a certain angel. With the rotating of the *X*, *Y* servomotor, the marking contents would appear, just as shown in Figure 2.

As we know, in the fonts, each character consists of a series strokes which is described by two points and a line. So the marking data of a character can be acquired from the font information. The data can be described as (*X*, *Y*, empty/real line), in which (*X*, *Y*) the 2-dimensional coordinates and the empty/real line will be set to 1 if the line is real and 0 if is the empty.

In addition, due to the different time response characteristics of dual-axis galvanometers, marking an arbitrary long line might look like a curve line, and it may not clear due to the fast responds of the servomotor. To solve this problem, discrete vectors were inserted into the original line. The number of discrete vectors depends on the setting resolution and the original line length. The insert discrete vectors are described as the structure below:

 struct markData;
 {int *x*;
 int *y*;
 int Flag;
 }point[*n*];

where *X* and *Y* are two-dimensional coordinates of the endpoint and FLAG is the status of the endpoint; if flag = 1, the point would be marked; otherwise, it will be skipped by turning off the laser.

## 3. Development of Laser Marking Control System Based on ARM9 and FPGA Processor

**Figure 3:**
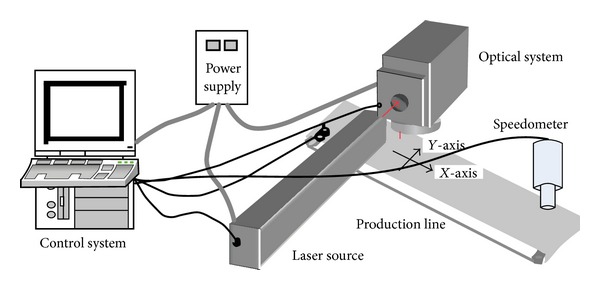
Structure diagram of the laser marking machine.

A typical laser marking system includes power supply, laser source, optical system, control system, and production line with speedometer, as shown in Figure 3. In delivery optics, the output laser beam reaches the galvanometers-based scanning system via pinhole, expander, then changes the moving direction by dual mirrors, and then it is focused by a f-theta lens at the surface of the workpiece to scribe the mark. The laser marking system should be capable of static and dynamic marking, so the speedometer is used to measure the speed of the production line. The control system should complete the following five functions. Firstly, it can control the power of laser beam through pulse mode. Secondly, it can control the scanner dual mirror to reach angle position simultaneously by sending out position voltage command to the servomotor. Thirdly, it should have human-machine interface so that the user can input the marking contents, for example, the text, image, or barcode, and can observe the operational status by LCD. Fourthly, it must be capable of sensing the speed of the production line by the speedometer. Lastly, the marking can be triggered automatically by the photoelectric switch which detects the coming of workpiece.

**Figure 4:**
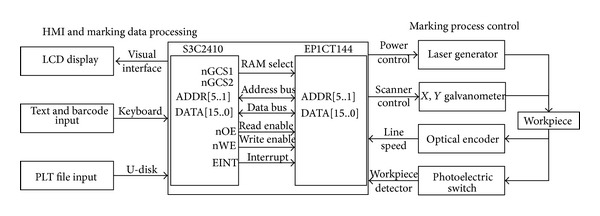
Schematic of the laser marking control system.

In order to execute these five functions fast, the controller is designed with dual-core, the ARM9 and FPGA processors. The functions are divided into two groups and are executed dividedly by the two processors. Just as shown in Figure 4, the ARM9 is responsible for the HMI and marking data processing and the FPGA is responsible for the marking process control.

### 3.1. The HMI and Marking Data Processing System Based on ARM9 Processor

**Figure 5:**
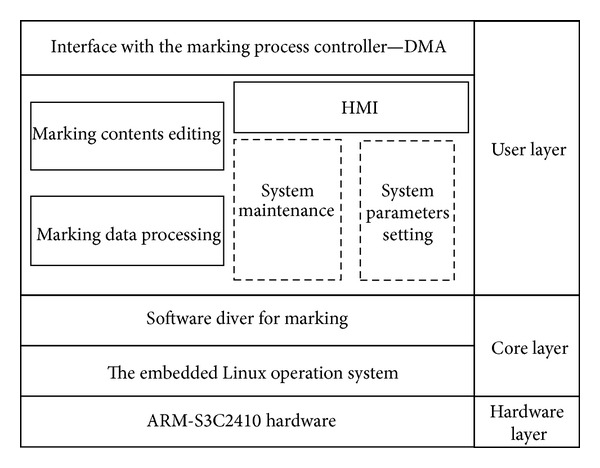
The software architecture of the HMI and marking data processing system.

The HMI and marking data processing task are designed to be completed by the main processor. It must have sufficient external resources, such as LCD interface, USB interface, and large storage capacity. Meanwhile, it must have fast enough clock speeds. Considering these factors, the S3C2410A running with LINUX2.4 embedded system is chosen to accomplish this task. The qt tools used for man-machine interface design, including information edit, LCD display, and the marking data processing, are installed on LINUX2.4. The marking contents are input by keyboard or U-disk and are displayed on the LCD. Then the description data of the marking contents are acquired from the font library, PLT files, or barcode generator tools. After that, the description data will be processed as marking point array. At last, they would be transmitted to the auxiliary processor by DMA communication mode for marking. The software architecture of the main controller is depicted in Figure 5 and was developed by C++ language.

### 3.2. The Marking Process Control System Based on FPGA Processor

**Figure 6:**
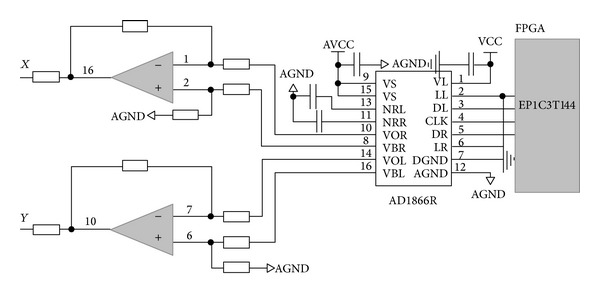
The schematic of galvanometer and laser power control circuit.

In the galvanometer control system, the *X*, *Y* galvanometers should be driven synchronously to guarantee the accuracy of the laser marking. FPGA processor with superior logic and parallel processing capabilities is capable of this task. So, the EP1C3T144, a kind of Cyclone II series device produced by ALTERA, is chosen for this application. In addition, the AD1866R, a kind of DA converter, and the OP270GS, a kind of precision differential amplifier, are used for position voltage generation and amplifier. The schematic circuit is described in Figure 6.

**Figure 7:**
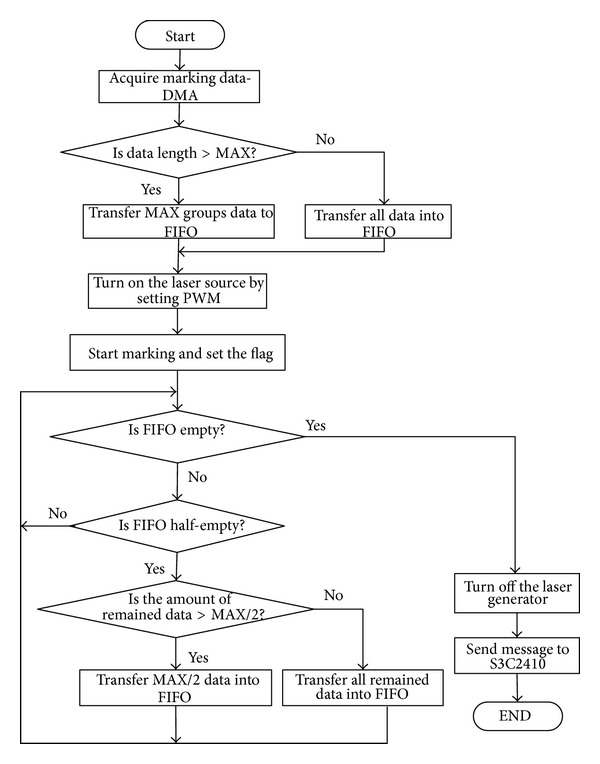
Software framework for the marking process control system.

In the marking process, the FPGA acquires the marking data which are described as the above mentioned structure marking data. When the marking process start, one set of *X*, *Y* digital value would be sent out to AD1866R and be converted to two voltages respectively, then they will be put into the controller to drive the two mirrors to predetermined angles. After a preset time, the second set of *X*, *Y* digital value would be sent out again to keep the mirrors rotating continuously. Simultaneously, if flag = 1, the laser source would be turned on; otherwise, it would be turned off.  Figure 7 shows the software framework for the marking control system. The VHDL language is used for this application.

## 4. Experiment System Setup

**Figure 8:**
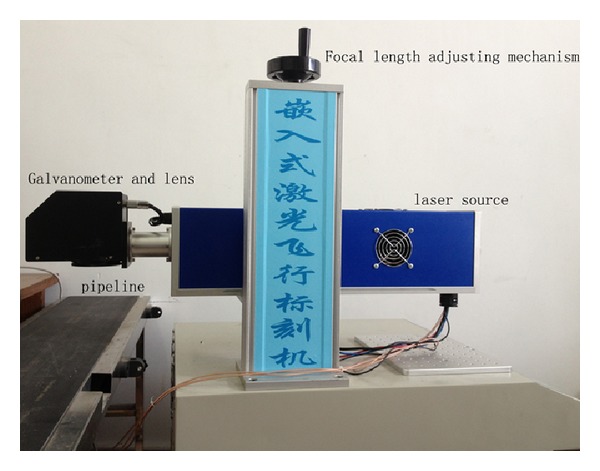
The laser marking experiment platform.

After the hardware and software were developed successfully, the embedded controller was used to control one laser marking machine. The machine is configured with the main components listed in Table 1. And Figure 8 shows one picture of the machine.

**Figure 9:**
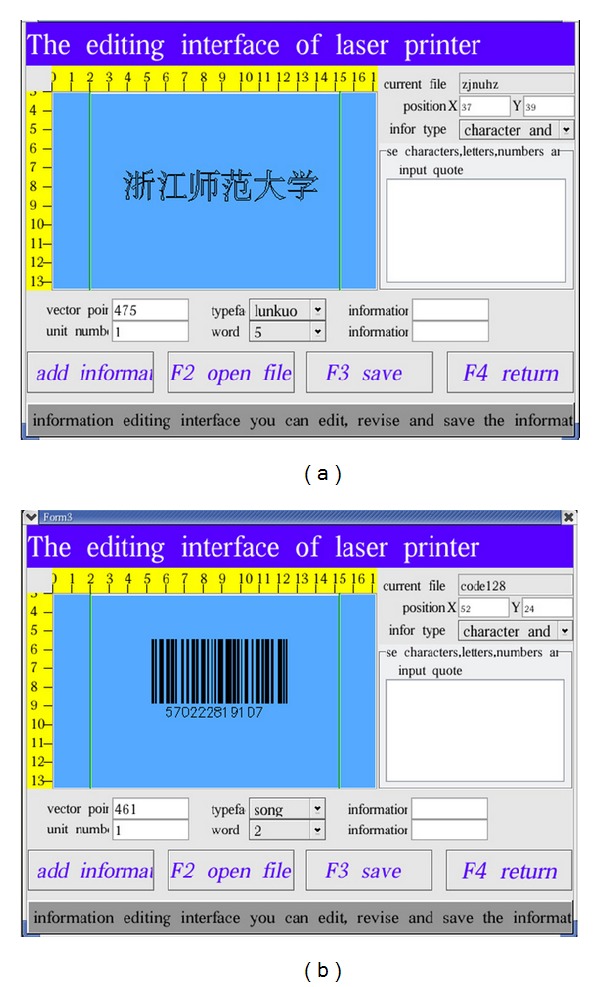
The HMI for editing marking contents ((a) text input or file open and (b) barcode generated).

**Figure 10:**
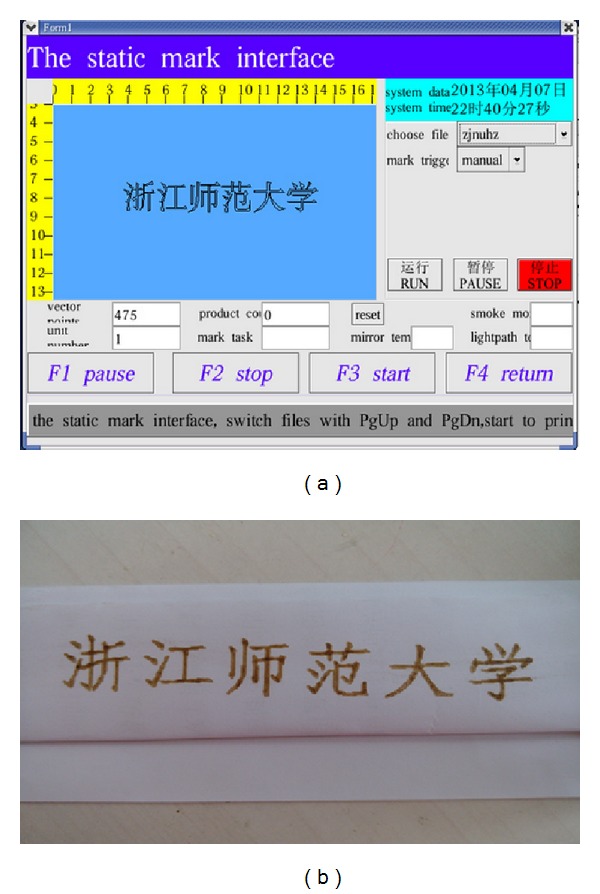
Pictures of the text input and marking ((a) text edit on HMI and (b) the marking result).

**Figure 11:**
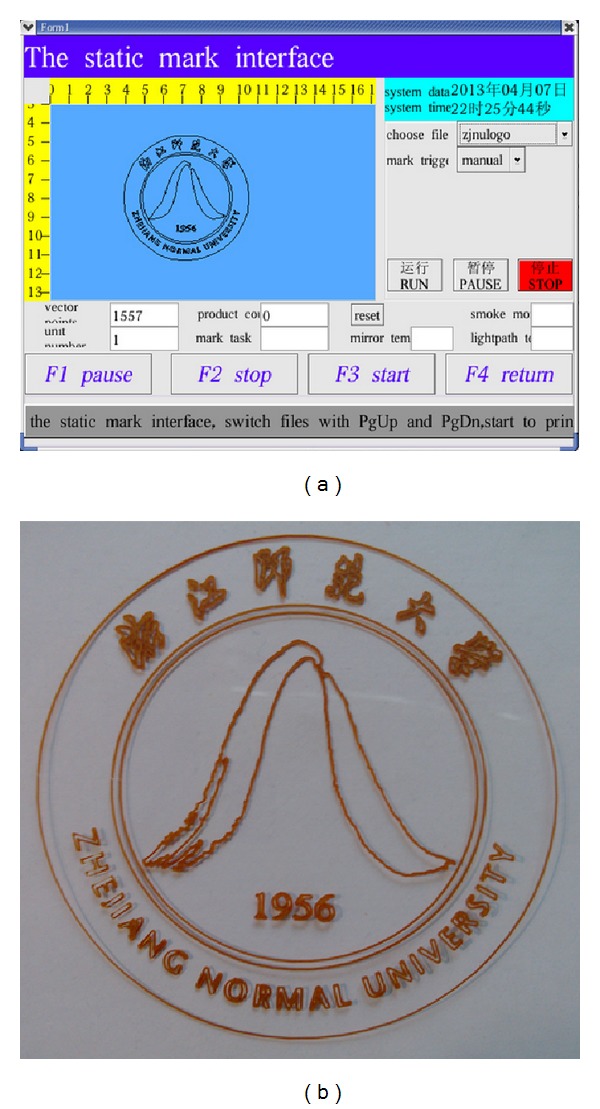
The logo of Zhejiang Normal University is marked ((a) the image prepared for laser marking and (b) the marking result).

**Figure 12:**
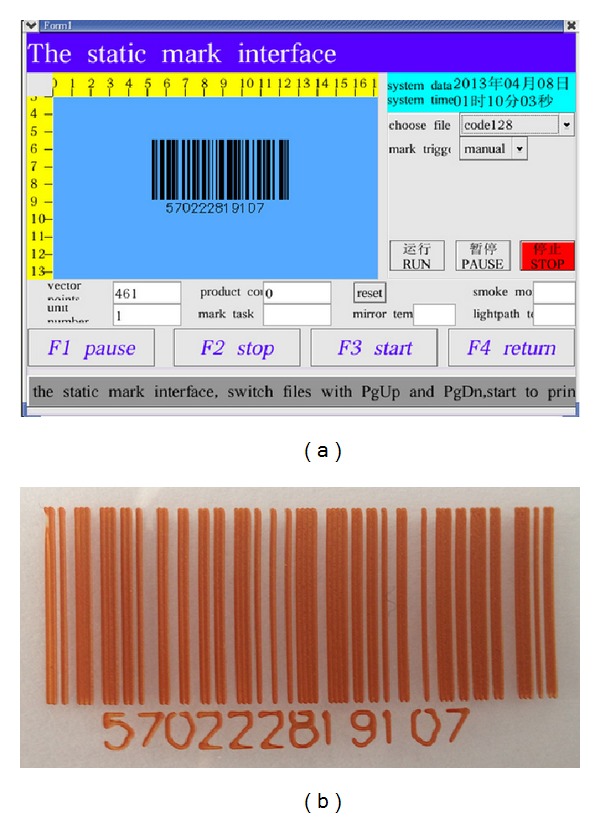
Pictures of the barcode marking ((a) the generated barcode and (b) the marking result).

The laser marking controller should be capable of marking text, image, and barcode on static and dynamic pipeline.  Figure 9 shows the HMI for marking contents editing. The text can be input directly by input method on HMI system. The image must be processed first by Photoshop (PS) software and then transferred to PLT file. Then, the PLT file can be loaded into the HMI system through the U-disc. The barcode is generated by software tool developed by our team members. Users only need to input the digital numbers, and the barcode will generate automatically. Figures 10, 11, and 12 show the marking results of text, image, and barcode on static working mode. Furthermore, the barcode can be recognized by recognition tools.

## 5. Discussion

The embedded laser marking controller based on ARM9 and FPGA is developed. The ARM9 processor is responsible for HMI and marking data processing and the FPGA is responsible for the marking process control. The division of work keeps the control system working with high speed. The C++ language and VHDL are chosen for ARM9 and FPGA software development. After establishing the hardware and software, a lot of experiments had been done. The results showed that the developed laser marking controller could achieve text, image, and barcode marking with high quality.

## Figures and Tables

**Table 1:** The parameters of the main component of the platform.

Devices	Model	Key parameters
Laser generator	Synrad 48-1 CO_2_ laser generator	Power: 10 W Wave length: 10.6 um

Galvanometer	TS8203 product by Beijing Century Sunny Technology Co., Ltd.	Supply voltage: ±24 V ± 10% Input voltage range: −10 V–+10 V

Controller	Self-developed	ARM9 + Linux2.4 + FPGA
